# Could Bryophagous Beetles (Coleoptera: Byrrhidae) Help Us Understand Bryophyte Taxonomy? Preferences within the *Hypnum cupressiforme* Hedw. Species Complex

**DOI:** 10.3390/plants10030469

**Published:** 2021-03-02

**Authors:** Petr Pyszko, Michaela Drgová, Stanislav Ožana, Ondřej Dorňák, David Rožek, Daniel Lee Číp, Vítězslav Plášek, Pavel Drozd

**Affiliations:** 1 Department of Biology and Ecology/Institute of Environmental Technologies, Faculty of Science, University of Ostrava, Chittussiho 10, 710 00 Ostrava, Czech Republic; michaela.drgova@osu.cz (M.D.); stanislav.ozana@osu.cz (S.O.); ondrej.dornak@osu.cz (O.D.); vitezslav.plasek@osu.cz (V.P.); pavel.drozd@osu.cz (P.D.); 2 Heyrovsky High School of Chemistry, Středoškolská 2854/1, 700 30 Ostrava, Czech Republic; 16rozekd@spsch.eu (D.R.); 18cipd@spsch.eu (D.L.Č.)

**Keywords:** bryophagy, bryophagous insects, herbivory, host preferences, *Hypnum cupressiforme*

## Abstract

Intrataxonomic differences in terms of angiosperm suitability for herbivorous insects stem from variables such as plant structure, palatability, and chemistry. It has not yet been elucidated whether these differences also occur in terms of the bryophyte’s suitability to bryophages. *Hypnum cupressiforme* Hedw. is a morphologically variable moss species frequently inhabited or fed by insects. In this investigation, we offered five morphotypes of *H. cupressiforme* to two bryophagous species of Byrrhidae (Coleoptera) to reveal whether the intrataxonomic variability affects beetles’ preferences. The morphotypes were offered with preserved and removed spatial structures. There were no significant differences in morphotype preferences when spatial structures were preserved, although during the daytime, the beetles moved from the *flat* morphotype to the *usual* and *turgid* morphotypes. The beetles preferred the *turgid* morphotype when the spatial structures were removed. The results suggest that the spatial structure variations in the *H. cupressiforme* complex are accompanied by different chemical, physiological, or microscopic morphological profiles that are recognized by the bryophagous insects. Phylogenetic and epigenetic analyses can reveal multiple differences within the *H. cupressiforme* complex. Their interconnection with information about the preferences of bryophagous insects can help us to elucidate which of these differences are ecologically relevant.

## 1. Introduction

There is a high level of variability in the diversity and abundance of insect species that associate with different plants [1]. Host preferences can be partially determined from the strength of the effect of top predators and parasitoids on the lower trophic levels, which varies with their abundance and efficiency [2,3], but mostly from host-related variables [4]. Plant variables such as the number of young leaves, leaf production, palatability, water content, plant height, nitrogen content, phenotypic variability, and secondary compounds determine the nutritional and mechanical properties of plants as hosts for herbivores [5,6,7,8,9]. The aforementioned differences in plant traits are local and can be linked with small-scale genetic differentiations among plant populations [10], which alongside the physical conditions of different habitats and the competition or presence of (mycorrhizal) fungi can be caused by the herbivores themselves [11,12,13,14]. Local antiherbivore defenses best demonstrate this genetic variability and geographically dependent phenological changes in leaf quality [15], and these result in a geographic mosaic for host plant suitability [16,17]. Variabilities in host plant suitability have been proven for herbaceous and woody plants, annuals, perennials, and aquatic species [10], but not yet for mosses.

*Hypnum cupressiforme* is a species that shows a large amount of morphological and ecological variability, mainly in European populations. This is because it is likely to have undergone genetic differentiation in Europe during the ice ages of the Pleistocene. Due to the East–West barrier of the Alps, the species survived in different refugia in southern Europe, where the populations were separated from each other for one to two hundred thousand years [18]. The high number of morphologically distinct forms is also reflected in the taxonomy, as some are occasionally classified as new species cf. [19,20]. Within the entire *Hypnum* genus which is sometimes considered highly polyphyletic, the *H. cupressiforme* forms quite a compact clade [21,22].

*H. cupressiforme* is one of the most frequent and dominant moss species in temperate regions, and it provides a suitable habitat for many invertebrate taxa [23,24]. *H. cupressiforme* may also have a high level of diversity and a high density of nematodes [25], small gastropods [26], tardigrades [23,27,28,29], chilopods [24], or collembolans [30,31]. Furthermore, it provides oviposition sites for spiders [32], hibernation sites for Ichneumonids [33], and is part of the diet of bryophagous insects including Tetrigidae (Orthoptera) [34], Chrysomelidae and Byrrhidae (Coleoptera) [35,36,37], the larvae of Cylindrotomidae (Diptera) [38] and Gelechiidae, Pyralidae, and Crambidae (Lepidoptera) [39,40], and also some mammals, such as *Microtus agrestis* (Linnaeus, 1761) [41]. *H. cupressiforme* also provides nesting material for ants, small rodents, and birds [42,43,44].

As most of the above references are from zoological studies, the specific morphotypes of the moss were not stated or discussed. The only exception was an investigation on the nesting materials used by three species of tits, which demonstrated high selectivity among *H. cupressiforme* morphotypes based on the width of the stems [45]. The variability of *H. cupressiforme* morphotypes is based mainly on their spatial structures, particularly on the size of their moss cushions, the length, width, branching patterns of the stems, or the spaces among the stems within the mats [19,20]. As moss species that form compact cushions retain moisture longer than species with open growth forms [46] and because this water retention is a crucial prerequisite for invertebrate microhabitats [47], the spatial structure features could play an important role in the selection of mosses by insects that use them frequently as shelters [48]. The spatial structures of mosses are also crucial for bryophagous insects, as the suitability of mosses as a microhabitat can outweigh their suitability as a host [49]. On the other hand, as the morphological variations may relate to genetic (or epigenetic) variations, the morphotypes may also differ in features other than spatial structure, such as their chemistry. However, these may not be separable based on their spatial structures in nonexperimental conditions.

In this study, for the first time on interactions between bryophages and bryophytes, we aimed to study the effect of intraspecific variability of bryophytes on bryophage’s preferences. We investigated whether bryophages discriminated between five common morphotypes of *H. cupressiforme* when their spatial structures were preserved. Furthermore, as the preferences in bryophagous insects could be caused or obscured by morphological differences, we conducted the experiment in parallel on mosses whose spatial structures were removed, to determine the variability caused primarily by nonstructural features. We aimed to determine whether there were differences in the preferences after removing the moss spatial structures, as this may suggest that *H. cupressiforme* morphotypes differed by more than their appearance from (not only) a bryophage’s point of view.

## 2. Materials and Methods

### 2.1. Experimental Design

As model bryophages, we chose two species from the family Byrrhidae (Coleoptera). Both species *Cytilus sericeus* (Forster, 1771) (n = 60) and *Byrrhus pilula* (Linnaeus, 1758) (n = 30) were collected by visual inspection of mosses and by individual hand sampling in September 2020 from Kozmice village in the Czech Republic (49.934° N, 18.166° E). We collected the moss mats of *H. cupressiforme* in September and October 2020 from three localities (1. Darkovičky village: 49.925° N, 18.181° E, 265 m.a.s.l., 2. Vítkov town: 49.799° N, 17.762° E, 480 m.a.s.l., 3. Krásná settlement: 49.558° N, 18.497° E, 925 m.a.s.l., all in the Czech Republic). The moss mats were classified into morphotypes differing mainly in spatial structure and determined by Vítězslav Plášek (bryologist, taxonomist), as previously described [19,20]. Based on their abundance and incidence, we chose five morphotypes:

1. The TURGID morphotype is characterized as having robust mats consisting of stems up to 5–8 cm in length and branches up to 3 mm wide. The free space among the individual stems in the mats is 0.3–1.5 cm. This morphotype grows most often on forest floors and in forest litter, and does not occur as an epiphyte on tree bark.

2. The USUAL morphotype corresponds to the description in most identification keys and is the most common morphotype for this species. It is characterized by medium-sized mats consisting of stems up to 3–5 cm in length and branches up to 2 mm wide. The free space among individual stems in the mats is 0.2–1.0 cm. This morphotype has no environmental preference for the substrate. It grows on forest floors, stones, and rock walls, but also as an epiphyte on the bark of deciduous and coniferous trees.

3. The FILIFORME morphotype is characterized by small slender mats consisting of nonbranching (or very rarely branching) stems up to 5 cm in length and up to 1 mm wide. The free space among the individual stems in the mats is 0.4–0.6 cm. This morphotype grows mostly on stones, boulders, and rock walls, and occurs as an epiphyte on tree bark. It is confined to vertical surfaces.

4. The RETICULATE morphotype is akin to the previous morphotype but differs as its stems are richly and distinctly branched. The stems are slender, up to 5 cm in length, and the branches are up to 1 mm wide. Free space among the individual stems in the mats is from 0.3–0.6 cm. This morphotype grows mostly on stones, boulders, and rock walls, and occurs as an epiphyte on tree bark. It is confined to vertical surfaces.

5. The FLAT morphotype is characterized by having medium-sized compressed-like mats consisting of stems up to 3 cm in length and branches up to 1.5–2 mm wide. Free space among the individual stems is limited by the flat habitat to 0.2 cm. This morphotype grows on the forest floor and boulders and does not occur as an epiphyte on tree bark. It is confined to horizontal surfaces.

We are aware of the high variability and taxonomic complexity of the genus *Hypnum* and therefore we were forced to rule out the possibility of incorrect identification of individual morphotypes. All morphotypes were studied in detail by a specialist and all used samples belong to *H. cupressiforme* (according to [50]), not to another of the many segregants of the traditionally conceived genus.

**Figure 1:**
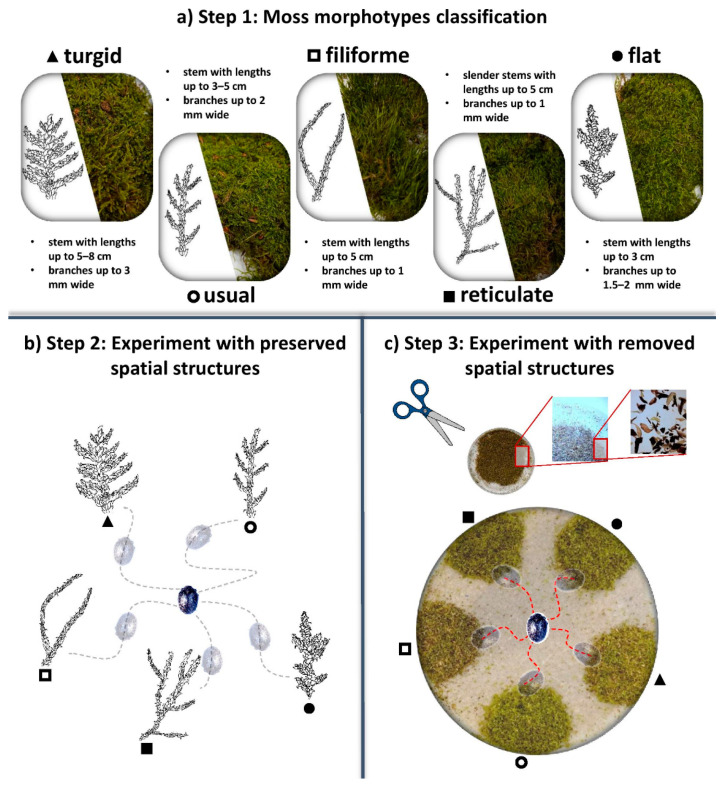
(**a**) Step 1: the samples of *H. cupressiforme* were classified into five morphotypes differing in spatial structure features (e.g., stems length or branches width as shown in the picture); (**b**) Step 2: the first group of beetles was presented with normal mosses with “preserved“ structures; (**c**) Step 3: the second group of beetles was presented with mosses very finely cut by a razor and thus with “removed“ spatial structures. In both cases, the beetle position was observed for 60 s after 1 h of exposition.

The beetles and mosses were held separately in plastic boxes under 12/12 light/dark cycles at 20 °C, and 70% humidity. After five days of acclimation, the individual beetles were repeatedly placed in circular plastic boxes (each beetle in one box) with the mosses arranged along the walls. The beetles were divided into two groups: the first group (*C. sericeus*, n = 30; *B. pilula*, n = 15) was presented with normal mosses or those with a “preserved structure”; the mosses in the second group (*C. sericeus*, n = 30; *B. pilula*, n = 15) were very finely cut by a razor (maximal particle sizes were checked for samples from each morphotype from each locality with a microscope to ensure that they had interquartile range = 0.79–0.96 mm), so that the spatial structure was removed and the moss matter was homogenized. The morphotypes were presented at the same weight (0.10 g) to each beetle. The morphotypes were randomly ranked in each box (replication without repetition) and the localities of their origin were also randomly selected (replication with repetition). After 1 h, beetle position was observed for 60 s and noted. In several cases, when the beetles chose more than one moss during the observation period, the preferences were divided equally between the visited mosses (Figure 1). The term preference indicated a choice of the moss as either a microhabitat or as part of their diet in an indistinguishable way, because the recognition of the phylloid fragments from the feces was not possible with sufficient certainty among the morphotypes. The experiment was repeated three times a day (08:00, 14:00, and 20:00) for 11–14 days. Beetles were kept on wet cotton wool and starved between the measurements. The presence of feces was checked, the mosses were moistened with a syringe, and new batches of mosses with preserved or removed structures were arranged into circular boxes each day after the last observation. For further analyses we used: (a) *C. sericeus* on the mosses with preserved structures (days = 14, n = 15; days = 11, n = 15); (b) *B. pilula* on the mosses with preserved structures (days = 11, n = 15); (c) *C. sericeus* on the mosses with removed structures (days = 14, n = 22), and (d) *B. pilula* on the mosses with removed structures (days = 14, n = 15). Beetles that died during the first 10 days of the experiment were discarded from further analyses.

### 2.2. Statistical Analysis

We analyzed data in R 4.0.2 using generalized estimating equation models with binomial distributions from the library “geepack” [51,52,53]. The dependent variable was the presence/absence of the beetles on a particular morphotype, the individual beetles were random effects, and autocorrelation structures were defined as “exchangeable”. To determine if the “normal” mosses with preserved structures were preferred differentially, we built the model with locality as a covariate, the morphotype of the moss as a main explanatory variable, and the day of the experiment, time of the controls, beetle genus, and their interactions with the morphotype of the moss as the other explanatory variables. The model was simplified by backward selection based on the rules of marginality to the final one with locality as a covariate and the morphotype of the moss, the time of the controls, and their interaction as explanatory variables. In the final model, the moss morphotypes were replaced according to their spatial structure features: stem length, branch width, minimal free space among branches, maximal free space among branches, ratio between maximal and minimal space, and propensity to epiphytism as ecological features. These modifications were compared with the original model using the quasi-likelihood information criterion (QICc) [54,55,56]. The models with ΔQICc ≤ 10 were considered as competitive with the best model. The best explanation was depicted in the plot using the “sciplot” library [57]. To determine whether mosses with removed structures were differently preferred, we used the same procedure, except that the final model contained locality as a covariate and the morphotype of the moss, the day of the experiment, and their interaction as explanatory variables. To evaluate whether there were potential differences in the preferences between the mosses with preserved or removed structures, we used the type of experimental design, moss morphotype, and their interactions as explanatory variables. The potential trends in the created plots were depicted using local polynomial regression fitting (loess) with a degree of smoothing = 2 [58].

## 3. Results

### 3.1. Preferences for Mosses When Structure Was Preserved

**Figure 2:**
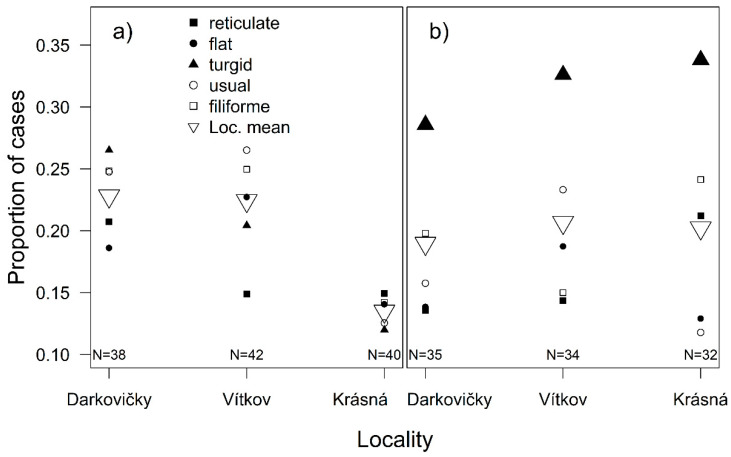
Proportion of cases when beetles chose a particular morphotype (*reticulate, flat, turgid, usual, filiforme*) of moss sampled from individual localities (Loc. mean = mean proportion of cases when beetles chose the mosses sampled from the locality), when the mosses had (**a**) preserved spatial structures or (**b**) removed spatial structures. The differences in proportion of cases when beetles chose a morphotype from individual localities were tested by generalized estimating equation models with binomial distribution. “N” denotes the number of beetles encountering a morphotype from a given locality.

**Figure 3:**
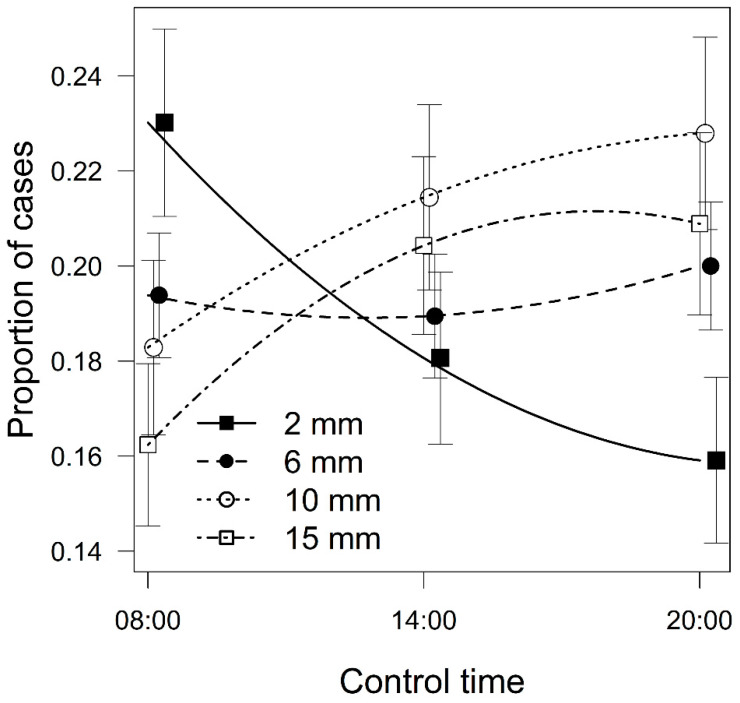
Proportion of cases when beetles chose mosses with preserved spatial structures with varying of the maximal free space among the stems (*flat* = 2 mm, *reticulate* = 6 mm, *filiforme* = 6 mm, *usual* = 10 mm, *turgid* = 15 mm) during the daytime (mean ± standard error, the trends are based on loess). The differences in proportion of cases when beetles chose a moss with given space among stems were tested by generalized estimating equation models with binomial distribution.

Preferences for the specific morphotypes were not significantly different (χ^2^ = 8.32, *P* = 0.081), and the differences between the individual localities were much greater (χ^2^ = 9.90, *P* = 0.007), with a lower preference for mosses from the mountain habitat (Figure 2a). During the day, the individual morphotypes were selected differentially (χ^2^ = 11.47, *P* = 0.022). The original model (with locality as a covariate and the morphotype of the moss, the time of the controls, and their interaction as explanatory variables) had the lowest QICc values in comparison to simplified models replacing the individual morphotypes with the spatial structure features. However, among the simplified models, the best explanatory variable was maximal free space among the branches (ΔQICc = 0.815), but three other models were also within ΔQICc ≤ 10 (Table 1). The model for maximal free space among the branches showed that, during the day, beetles moved significantly (χ^2^ = 8.08, *P* = 0.004) from the mosses with smaller free spaces between their branches (type *flat*) to the mosses with larger free spaces (type *usual* and *turgid*), whereas the preferences for the morphotypes with medium free spacing between the branches (type *filiforme* and *reticulate*) remained stable (Figure 3).

### 3.2. Preferences for Mosses When Structure Was Removed

**Figure 4:**
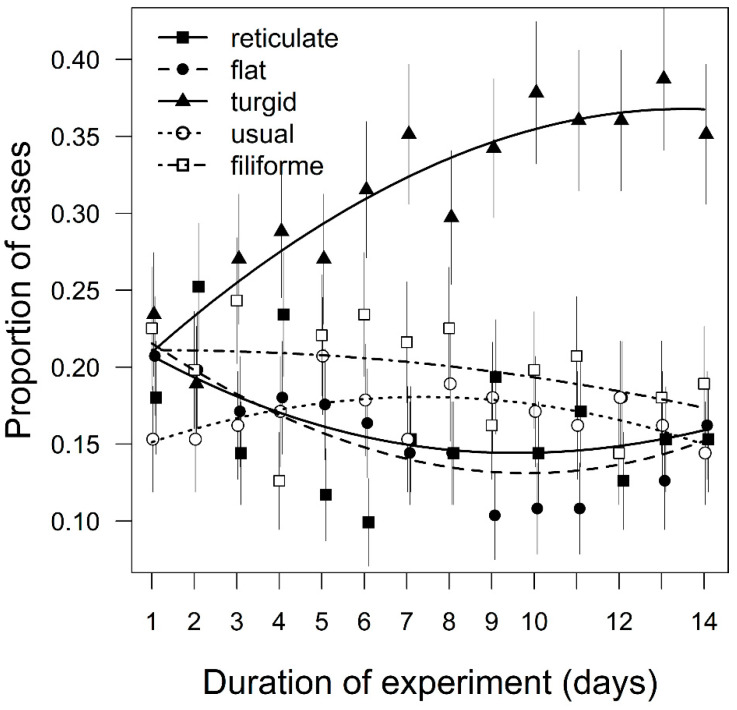
Proportion of the cases when beetles chose moss morphotypes (*reticulate, flat, turgid, usual, filiforme*) with removed spatial structures on each day of the experiment (mean ± standard error, the trends are based on loess). The differences in proportion of cases when beetles chose a particular morphotype were tested by generalized estimating equation models with binomial distribution.

Preferences for specific morphotypes of the moss differed significantly (χ^2^ = 121.90, *P* < 0.001), and the most *turgid* morphotype was the most popular. The differences at the level of localities were not significant (χ^2^ = 5.10, *P* = 0.078), although specific morphotypes were preferred differentially, based on the locality of their origin (χ^2^ = 30.50, *P* < 0.001), but the *turgid* morphotype was the most preferred regardless of locality (Figure 2b). During the experiment, the preferences for morphotypes changed (χ^2^ = 22.10, *P* < 0.001), as the preference for the *turgid* morphotype increased (Figure 4). The original model that explained variability according to individual morphotypes had the lowest QICc. None of the simplified models were within ΔQICc ≤ 10 (Table 1).

### 3.3. Effects of Treatment

**Figure 5:**
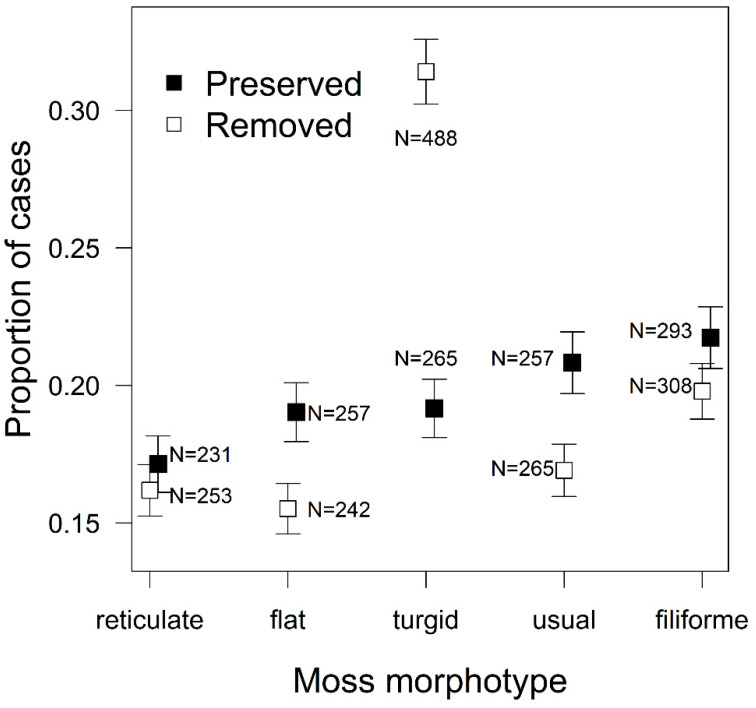
Proportion of cases when beetles chose the different moss morphotypes when the mosses had “preserved” or “removed” spatial structures (mean ± standard error). The differences in proportion of cases when beetles chose a particular morphotype were tested by generalized estimating equation models with binomial distribution. “N” denotes the number of positive cases when beetles chose a particular morphotype.

There were significant differences in the preferences for the different morphotypes (χ^2^ = 74.90, *P* < 0.001; Figure 2), and significant differences between the treatments (χ^2^ = 56.00, *P* < 0.001). The *turgid* morphotype was in the middle of the preference rank when mosses with preserved structures were offered, but was the most preferred morphotype when the structure of the mosses was removed (Figure 5).

## 4. Discussion

When the spatial structures were preserved, we did not find that the bryophagous beetles had any significant preferences for the individual *H. cupressiforme* morphotypes, but there was significant avoidance of the mosses from the mountain locality, regardless of morphotype. In contrast, when the spatial structures were removed, we found a strong preference for the *turgid* morphotype, regardless of the moss origin. In this investigation, we did not differentiate between situations where the moss was selected as a habitat or as part of the beetle’s diet. Observation for grazing may not provide direct evidence of further feeding. The bryophyte species can be identified easily based on the structures of the phylloid fragments, including those in the gut or feces [59], but this was not possible at the intraspecific level. On the other hand, the diet of the adults of both beetle species consisted exclusively of mosses, including *H. cupressiforme* [37]. This was because during the experiment they did not have access to any other source of food, and all beetles continuously produced feces, suggesting that they frequently fed on the mosses. Moreover, our experiment with a removed moss spatial structure was designed to avoid the bias caused by the selection of the mosses as a microhabitat. Despite this reasoning, the term preference was used in the manuscript as it was not possible to distinguish if the choice of moss was as a microhabitat or a diet.

### 4.1. Preferences for Mosses When Structure Was Preserved

The preferences for the specific morphotypes did not differ significantly but changed during the daytime. Beetles moved from mosses with smaller amounts of free space among their branches to mosses with larger amounts of free space. This indicates that although the preferences among the specific morphotypes did not differ overall, the beetles perceived them to be different. Based on the free space among branches, the morphotypes may differ in moisture retention, which could affect the palatability and microhabitat preferences [46,60,61]. Furthermore, the experimental manipulation of the beetles during the day may cumulatively have disturbed them, possibly simulating the threat of predation. Mosses serve as relatively useful shelters from a large number of terrestrial [38,48,62,63] as well as aquatic [64,65] predators, but some mosses may provide better protection against predators than others [66]. With respect to the previous conclusions, we can assume that the beetles may recognize various morphotypes as being differently suitable hiding places.

There was a great difference in the preferences at the level of individual localities, with low preferences for mosses from the Krásná locality. As this locality was at a higher altitude than the others (approx. 925 m.a.s.l.), it is possible that the mosses sampled there differed slightly in spatial shape, color, or some other features that were not considered. The environment can strongly affect the spatial structures of bryophytes, even in the same species. The water flow (for mosses growing on rocks and trunks), the amount of light, the length of the period with snow, or the direction and strength of the wind significantly contributes to the resulting spatial shape of the moss cushions [67]. Along the altitudinal gradient, plants also differed in UV absorbance and related morphological traits, including stunted growth and the formation of “sun leaves”, with higher concentrations of UV-B radiation absorbing pigments, higher specific leaf weight, leaf thickness, and leaf hair density [68,69,70,71]. The effects of the UV radiation on pigment composition, or physiological and morphological characteristics, has also been reported for mosses [72,73,74,75]. Along with altitude, the compositions and concentrations of the essential oils and other volatile organic compounds may also vary in plants [76,77], including *H. cupressiforme* [78]. Thus, it is possible that all morphotypes of *H. cupressiforme* from the mountain habitats contained lower amounts of attracting or conversely higher amounts of repellent volatiles. Furthermore, with increasing altitude, there is a decline in the concentrations of organic pollutants in the mosses [79], which are generally toxic to plants [80], and this may therefore affect their attractiveness and palatability to herbivores.

### 4.2. Preferences for Mosses When Structure Was Removed

Surprisingly, after the removal of the spatial structures, there were very significant preferences among the morphotypes, with the *turgid* morphotype being the most preferred. Closely related bryophyte species can have very different levels of preference for bryophagous insects [81], and in vascular plants even different host plant genotypes within a species can vary in their preference [17]. The *H. cupressiforme* complex showed substantial intraspecific variation. Frahm [18] pointed out that *H. cupressiforme sensu stricto* consists of several different phenotypes and potentially even genotypes, which may be distinguished as a high number of separate species. In addition to morphological differences, they also have different ranges and ecological preferences. Furthermore, the whole *Hypnum* genus was highly polyphyletic, and many unrelated populations were masked by convergent morphological evolution [21]. Thus, morphological species concepts without molecular analysis may be misleading in the *H. cupressiforme* complex [82].

The differentially preferred morphotypes may also show variability in their microscopic morphological traits not removable by destroying the moss spatial structures, such as cell size and cell wall thickness. Facultative bryophages may experience grinding of the mandibles when feeding on mosses [83]. Thus, bryophages may choose among mosses based on their cell wall thickness. The differential preferences among the hosts can also be based on physiology [84]. Sardans and Peñuelas [85] found that in *H. cupressiforme*, almost 70% of the variation in the moss elemental concentrations was explained by drought. Drought thereby changes moss stoichiometry, which could also affect the palatability and moss–herbivore relationships [85]. Herbivore pressure is generally higher in water-stressed plants due to their higher leaf nitrogen contents and better palatability [60,61]. The water retention in mosses decreases with decreasing density of the cushions, and species that form open growth forms retain moisture for a shorter period than species that form compact cushions [27,46]. Thus, the higher preference for the *turgid* morphotype by the bryophages should be expected, as this morphotype has the largest space among its stems [19,20], and consequently the most water-stressed physiology among the tested morphotypes. On the other hand, the suitability of the mosses as a microhabitat for the bryophagous insects outweighs their suitability as a diet [49]. Mosses with open growth forms may not be preferred as microhabitats [47], which may explain why the *turgid* morphotype was not the most preferred when the spatial structure was preserved.

Furthermore, the preferences of the mosses may be affected by chemical defenses [63]. *H. cupressiforme* has the capacity to produce secondary compounds with strong antibacterial, antifungal, and insecticidal effects [86,87]. This capacity may differ among the morphotypes, but our knowledge of the substance composition of the mosses is still poorly understood [88]. The moss canopy spatial structure may also be an important parameter in shaping microbial diversity [89]. As the fungal or bacterial microbiota of the Byrrhidae may be related to their diet processing and survival [90,91], the microbiota of the mosses (shaped by their spatial structures and retained after structure removal) may also be an important factor for the bryophage’s preferences. The hypothesis of favorable physiological, chemical, or microbiological profiles of the *turgid* morphotype could be supported by the increasing preferences for this morphotype during the days of the experiment, indicating beetles possible learning or physiological changes of this morphotype, which differed from the other morphotypes, but occurred regardless of the locality of origin. Moreover, none of the simplified models based on spatial structure traits could explain the differences in moss morphotype preferences when the spatial structures were removed.

### 4.3. Effects of the Treatment

We found an apparent discrepancy in the preferences for mosses with preserved and removed spatial structures, with a strong increase in preference for *turgid* morphotypes after the spatial structures were removed. This difference could be enhanced by the treatment itself. The removing of moss spatial structure by razor may release volatile secondary compounds whose concentrations may vary within the *H. cupressiforme* [78], and may attract or conversely repel the bryophages, in quantities greater than in normal conditions. Thus, we hypothesized that the *turgid* morphotype differs from the other morphotypes in terms of its physiology, chemistry, or other traits, but that its spatial structure obscured this pattern under normal conditions. These differences may be based on genetics. Moreover, epigenetic regulatory mechanisms can facilitate in plants the changes in gene activity and gene expression patterns, resulting in a high degree of phenotypic plasticity. Epigenetic factors have emerged as relevant modulators of plants’ responses to the environment, both abiotic stress or biotic interactions. However, the links between epigenetic and phenotypic variation in this context remain poorly studied [92,93].

This study is an evaluation of the pilot results. However, the research will continue in the near future. We will focus on detailed differences in anatomy and morphology of the used morphotypes. For differentiation, SEM photographs of leaf surface structures will be made, and anatomical leaf features will be compared in detail, including cell size and cell wall thickness. In order to find a specific explanation of the preference of individual morphotypes by insects, we intend to also make detailed chemical analyzes of all morphotypes using High Performance Liquid Chromatography—HPLC. We have used this method in the past on species of the moss genus *Orthotrichum* and in the case of *Hypnum* it could achieve good results.

## 5. Conclusions

Despite the slight differences in moss morphology, the bryophagous insects were able to recognize the heterogeneity among the morphotypes and populations of the *H. cupressiforme*. Surprisingly, the preferences among the morphotypes were significant after the removal of the spatial structure differences, and these differences were probably accompanied by differing microscopic morphological, physiological, chemical, or microbial profiles, resulting from different genetic bases, or from the original spatial structures. However, we can only speculate on the sources of these differences. Within the forest vegetation of the Czech Republic, where sampling was performed, two additional morphotypes not involved in this study were observed in mountain coniferous forests. Based on our rich field experience, we are able to also distinguish at least five other morphotypes of *H. cupressiforme* in the nonforest vegetation such as meadows, exposed rocks, shifting sands or salt marshes. Thus, more detailed phylogenetic, physiological, and chemical analyses of the various populations of *H. cupressiforme* or of the whole *Hypnum* genus are required. Moreover, some experts consider that the individual variations do not correspond to the genetic basis and are only the consequence of phenotypic plasticity. Thus, at least some morphotypes may be based on an identical genetic basis but with differing epigenetic profile caused—i.e., by the microhabitat conditions—and accompanied by the production of various volatiles and other secondary compounds.

Consequently, a combination of the phylogenetic and epigenetic analyses could reveal multiple differences among *H. cupressiforme* morphotypes. The interconnection of this information with the information about the preferences of bryophagous insects within *H. cupressiforme* complex could yield surprising results regarding *Hypnum* taxonomy, evolution, and interactions with bryophages. Thus, we urge all bryophagous and bryobiontic insect ecologists to indicate the morphotypes of the *H. cupressiforme* in case of their association with insects, as insect preferences among morphotypes may vary greatly and be ecologically relevant.

## Figures and Tables

**Table 1:** Quasi-likelihood information criterion (QICc) evaluations for the individual models for mosses with preserved/removed structures based on their individual morphotypes or their various morphological or ecological features.

Structure	Individual Morphotype (QICc)	Stem Length (QICc)	Branch Width (QICc)	Minimal Fsab ^1^ (QICc)	Maximal Fsab ^1^ (QICc)	Variance in Fsab ^1^ (QICc)	Propensity to Epiphytism (QICc)
Preserved	6643.60 ***	6649.24 *	6654.36	6652.37 *	6644.42 **	6645.96 *	6655.56
Removed	7599.20 ***	7631.35 *	7678.90	7753.61	7640.81	7711.45 *	7742.63

^1^ free space among branches. *** indicates the best model; ** indicates the best simplifying model; * indicates models with a ΔQICc ≤ 10.

## Data Availability

The data presented in this study are openly available in Figshare [doi: 10.6084/m9.figshare.13614131].
